# Quantitative phosphoproteomics to unravel the cellular response to chemical stressors with different modes of action

**DOI:** 10.1007/s00204-020-02712-7

**Published:** 2020-03-18

**Authors:** Bharath Sampadi, Alex Pines, Stephanie Munk, Branislav Mišovic, Anton J. de Groot, Bob van de Water, Jesper V. Olsen, Leon H. F. Mullenders, Harry Vrieling

**Affiliations:** 1 Department of Human Genetics, Leiden University Medical Center, P.O. Box 9600, 2300 RC Leiden, The Netherlands; 2 Novo Nordisk Foundation Center for Protein Research, Proteomics Program, Faculty of Health and Medical Sciences, University of Copenhagen, Blegdamsvej 3b, 2200 Copenhagen, Denmark; 3 Division of Drug Discovery and Safety, Leiden Academic Centre for Drug Research, Leiden University, Einsteinweg 55, 2333 CC Leiden, The Netherlands; 4 Department of Genetics, Research Institute of Environmental Medicine (RIeM), Nagoya University, Nagoya, Japan

**Keywords:** Phoshoproteomics, Stress signaling, Cisplatin, Etoposide, Cyclosporine A, Diethyl maleate

## Abstract

Damage to cellular macromolecules and organelles by chemical exposure evokes activation of various stress response pathways. To what extent different chemical stressors activate common and stressor-specific pathways is largely unknown. Here, we used quantitative phosphoproteomics to compare the signaling events induced by four stressors with different modes of action: the DNA damaging agent: cisplatin (CDDP), the topoisomerase II inhibitor: etoposide (ETO), the pro-oxidant: diethyl maleate (DEM) and the immunosuppressant: cyclosporine A (CsA) administered at an equitoxic dose to mouse embryonic stem cells. We observed major differences between the stressors in the number and identity of responsive phosphosites and the amplitude of phosphorylation. Kinase motif and pathway analyses indicated that the DNA damage response (DDR) activation by CDDP occurs predominantly through the replication-stress-related *Atr* kinase, whereas ETO triggers the DDR through *Atr* as well as the DNA double-strand-break-associated *Atm* kinase. CsA shares with ETO activation of CK2 kinase. Congruent with their known modes of action, CsA-mediated signaling is related to down-regulation of pathways that control hematopoietic differentiation and immunity, whereas oxidative stress is the most prominent initiator of DEM-modulated stress signaling. This study shows that even at equitoxic doses, different stressors induce distinctive and complex phosphorylation signaling cascades.

**Electronic supplementary material:**

The online version of this article (10.1007/s00204-020-02712-7) contains supplementary material, which is available to authorized users.

## Introduction

Cells are equipped with versatile physiological stress responses to prevent hazardous consequences resulting from exposure to chemical insults of endogenous and exogenous origin. In case these stress responses fall short in their protective function, cells may die or become derailed with disease development as a potential outcome. Activation of cellular stress responses entails signaling and effector stages with the induction of post-translational modifications (PTM) of specific proteins and directed transcriptional alterations, respectively, as main molecular mechanisms. While stressor-induced changes at the gene expression level have been the focus of numerous studies, the upstream signaling events mediated by PTMs have scarcely been investigated at a proteome-wide level. Among the various types of PTMs that are involved in cellular stress responses, one of the most frequent modifications is the reversible and dynamic phosphorylation of proteins at specific serine (pS), threonine (pT) and tyrosine (pY) residues (Bennetzen et al. 2010; Olsen et al. 2010). Stressor-inflicted DNA damage, alterations in chromatin conformation and changes in DNA metabolism can all activate protein kinases that phosphorylate distinct subsets of proteins (Choudhary and Mann 2010; Polo and Jackson 2011; Warmoes et al. 2013). It has been estimated that almost all proteins in mammalian cells are phosphorylated at some point during their expression with over 75% detected with current mass spectrometry technology (Sharma et al. 2014; Humphrey et al. 2015, 2018). Stable isotope labeling of amino acids in cell culture (SILAC) is one of the most accurate quantification techniques for phosphoproteomics. While SILAC enables small-scale multiplexing of samples, it suffers from a decrease in total phosphopeptide ratio quantifications due to repeated sequencing of phosphopeptide isotope variants (Hogrebe et al. 2018).

In this study, we used SILAC-based phosphoproteomics to investigate to what extent toxic stressors with different modes of action evoke common and stressor-specific signaling responses when administered at equitoxic doses to in-vitro cultured cells (Pines et al. 2011). We focused on four stressors with distinctive properties and modes of action that do not require bioactivation, i.e. cisplatin (CDDP), etoposide (ETO), diethyl maleate (DEM), and cyclosporine (CsA). CDDP induces intra- and inter-strand DNA cross-links as well as mono adducts that can interfere with transcription and replication (Todd and Lippard 2009; Wang and Lippard 2005). The topoisomerase II inhibitor ETO forms a ternary complex with topoisomerase II and DNA leading to the formation of DNA double-strand breaks (DSBs) in transcribed genes and at replication forks (Deweese and Osheroff 2009; Vesela et al. 2017). Exposure to the pro-oxidant DEM generates a rapid depletion of cellular GSH and as a consequence leads to enhanced levels of reactive oxygen species (ROS) that may induce lipid peroxidation (Tirmenstein et al. 2000; Weber et al. 1990). The immunosuppressive agent CsA is a non-genotoxic carcinogen in humans (Olshan et al. 1994) and mouse (Kesteren et al. 2009) and acts as an inhibitor of the protein phosphatase calcineurin (Leyking et al. 2015; Matsuda and Koyasu 2000). Phosphoproteome analyses allowed us to identify common and stressor-specific phosphorylation signaling pathways for each of the four stressors and provide insights into the nature of the cellular processes that are (in)activated to minimize cellular toxicity by stressors with different modes of action.

## Materials and methods

### Cell culture and treatment

Cell culture and treatment of wild-type B4418 mouse embryonic stem (mES) cells were essentially performed as previously described (Pines et al. 2011). Sub-confluent cultures of mES cells were exposed to either CDDP (5 µM) (Accord, 1 mg/ml), CsA (20 µM) (Sigma, Catalog No. 30024-25 mg), DEM (150 µM) (Sigma, Catalog No. D97703-100G) or ETO (0.5 µM) (Sigma, Catalog No. E1383-25 mg) by adding the drug directly to the culture medium and cells were incubated for different periods after administration (2, 4, 6 and 24 h). To inhibit *Atm *and* Atr* functions, cells were treated with the *Atm* inhibitor Ku-55933 (10 µM) (Selleckchem, Catalog No. S1092) and the *Atr* inhibitor VE-821 (1 µM) (Selleckchem, Catalog No. S8007), respectively. Cell culture media was prepared by mixing 55 ml dialyzed FBS (Thermo Scientific Catalog No. 88440), 5.5 ml Glutamax (Life Technologies, Catalog No. 35050-038), 5.5 ml PenStrep (Life Technologies, Catalog No. 15140-122), 1.25 ml β-mercaptoethanol (Gibco, Catalog No. 31350010) and 55 μl leukemia inhibitory factor (LIF) (Millipore, Catalog No. ESG1107) to 500 ml DMEM media minus l-lysine and l-arginine (Thermo scientific, Catalog No. 89985). An appropriate amount of medium was prepared for labeling cells using 10 mg arginine, 20 mg lysine and 20 mg proline for 100 ml medium. *Light SILAC:*l-arginine (Arg 0) (Cambridge Isotope Laboratories Inc, Catalog No. ULM-8347) and l-lysine:2HCl (Lys 0) (Cambridge Isotope Laboratories Inc, Catalog No. ULM-8766), *Medium SILAC:*l-arginine—U-^13^C_6_ (Arg6) (Cambridge Isotope Laboratories Inc, Catalog No. CLM-2265-H) and l-lysine—U-4,4,5,5,-D4 (Lys 4) (Cambridge Isotope Laboratories Inc, Catalog No. DLM-2640) and *Heavy SILAC:*l-arginine—U-^13^C_6_, 99%; U-^15^N_4_, 99% (Arg 10) (Cambridge Isotope Laboratories Inc, Catalog No. CNLM-539) and l-lysine—U-^13^C_6_, 99%; U-^15^N_2_, 99% (Lys 8) (Cambridge Isotope Laboratories Inc, Catalog No. CNLM-291). Furthermore, l-proline (Cambridge Isotope Laboratories Inc, Catalog No. ULM-8333) was added to all SILAC media to prevent arginine to proline conversion, which sometimes makes interpretation of MS data more difficult. Cells were cultured in an isotopically labeled medium for 6–7 cycles before performing experiments for MS analysis. During passages, the medium was decanted, cells were washed with PBS (Dulbecco’s PBS without calcium, magnesium, and phenol red 500 ml; Life Technologies, Catalog No. 14190086) and trypsinized using TrypLE (100 ml; Life Technologies, Catalog No. 12604-013).

### Phosphoproteomic sample preparation

Protein sample collection, phosphopeptide enrichment, and cleanup were performed according to previously published procedures (Pines et al. 2011). Briefly, cells were collected after 4 h of drug treatment by trypsinization and cell pellets were lysed for 30 min in lysis buffer [8 M urea, 50 mM Tris (pH 8.1), 75 mM NaCl, 1 mM MgCl_2_, 500 Units benzonase (Novagen, Catalog No. 71205-3), 1 tablet complete mini protease inhibitor, EDTA-free (Roche, Catalog No. 11836170001), 100 µl Phosphatase Inhibitor Cocktail 3 (RT 1 h before use; Sigma, Catalog No. P0044-5ML) and 100 µl Phosphatase Inhibitor Cocktail 2 (Sigma, Catalog No. P5726-5ML)]. Samples were then centrifuged (13,000 rpm 15 min) to remove the cell debris. Protein concentrations were estimated (Qubit Protein Assay kit; Invitrogen, Catalog No. Q33212) and 10 mg of proteins per sample was taken for protein digestion. After reduction (2.5 mM dithiothreitol (DTT) for 25 min at 60 °C), alkylation [7 mM iodoacetamide for 15 min at room temperature (RT) in dark] and quenching (2.5 mM DTT for 15 min at RT), samples were diluted eightfold with 25 mM Tris (pH 8.1) − 1 mM CaCl_2_. Proteins were digested with 100 µg trypsin (sequencing grade 100 µg; Promega, Catalog No. V5111) for 15 h at 37 °C and the reaction was stopped by adding trifluoroacetic acid (TFA) to a final concentration of 0.4%. Following centrifugation (3200 rpm for 5 min), supernatants containing tryptic peptides were loaded onto tC_18_ cartridges (Sep-Pak Vac 1 cc 100 mg; Waters, Catalog No. WAT036820). Peptides were desalted (0.1% acetic acid), eluted [0.1% acetic acid (ACA) and 30% acetonitrile (ACN)], lyophilized and fractionated on a 9.4 by 200-mm 5 µm particle polysulfoethyl, a strong cation exchange (SCX) column (PolyLC) at 1 ml/min using a 70-min gradient from 0 to 75 mM KCl, with 350 mM KCl for 38 min in 5 mM KH_2_PO_4_ (pH 2.65) and 30% acetonitrile. Initially, 18 fractions were collected and during desalting, the last 8 fractions were combined into 4 leading to a total of 14 fractions which were then lyophilized. Each of these 14 fractions were then dissolved in buffer A (300 mg/ml lactic acid, 80% ACN, 0.1% TFA) and loaded on to titanium dioxide (TiO_2_) columns (TopTip 1–10 µl; Glygen Corporation, Catalog No. TT1TIO96) prewashed with elution buffer (15 mM NH_4_OH, pH 10.5), equilibration buffer (0.1% TFA) and buffer A. Samples were then washed with buffer A and B [80% ACN (v/v), 0.1% TFA (v/v)]. Phosphopeptides were eluted from the columns with elution buffer and collected in Eppendorf tubes with equal volumes of 2% TFA. Phosphopeptides were subsequently loaded onto Stage Tip C_18_ column (20 μl; Proxeon, Catalog No. SP201) equilibrated with methanol, buffer B and 0.1% TFA. Next, they were desalted (0.1% TFA), eluted (buffer B) and lyophilized and stored at − 80 °C.

### High-performance liquid chromatography (HPLC) and mass spectrometry (MS) measurements

HPLC and MS measurements were performed as described below. Phosphopeptides were resuspended in 5% acetonitrile (ACN) in 0.1% trifluoroacetic acid for downstream MS analysis. All enriched samples were analyzed by online LC–MS/MS on an EASY-nLC system (Thermo Scientific, Odense, Denmark, Catalog No. LC120) interfaced with a Q-Exactive orbitrap mass spectrometer (Thermo Electron, Bremen, Germany, Catalog No. IQLAAEGAAPFALGMAZR) through a nano-electrospray ion source. All peptides were auto-sampled and separated on a 15 cm column (75 μm internal diameter) packed in-house with 3 μm C18 beads (Reprosil-AQ Pur, Dr. Maisch, Germany, Catalog No. r13.aq). Peptides were separated over 80 min by a linear gradient from 8 to 60% of acetonitrile (ACN) in 0.5% acetic at a flow rate of 500 nl/min till 10% ACN, then 250 nl/min up to 50% ACN and then 500 nl/min for the remaining gradient. Spray voltage was 2 kV, S-lens RF level at 50%, heated capillary at 275 °C, no sheath or auxiliary gas flow. MS was performed in a data-dependent acquisition mode where the 12 most intense, multiply charged peaks were chosen for fragmentation after acquiring each full spectrum. Dynamic exclusion was used set to 30 s to avoid picking peaks more than once. Full scans were acquired at a mass range of 300–1750 *m*/*z* with a resolution of 70,000 at *m*/*z* 200, with target 3 × 10^6^ based on predictive AGC from the previous full scan, with a maximum fill time of 30 ms. Fragmentation was performed using Higher energy Collisional Dissociation (HCD) with a normalized HCD collision energy 25% and acquired with the target set to 3 × 10^6^, with max injection time of 120 ms at 35,000 resolution with an isolation window of 2 *m*/*z*.

### MS data analysis

The raw MS data were processed in MaxQuant (version 1.5.7.4) (FDR < 0.01 at the protein, peptides, and modifications levels) using the default settings with the following minor changes: SILAC triplet was activated (light = default; medium = Lys4; Arg6; heavy = Lys8; Arg10). Acetylation (Protein N-term), oxidation (M) and phosphorylation (STY) were selected as variable modifications and carbamidomethylation (C) was selected as a fixed modification. A minimum peptide length of six amino acids was set and “match between runs” (MBR) was enabled with the default value. A reverse sequence database was used to identify proteins and peptides by the inbuilt Andromeda search engine using the Mouse Uniprot FASTA database (November 2016). “ReQuantify” (REQ) was switched on. Further downstream analyses were performed using Perseus (version 1.5.5.3), Microsoft Excel (2016), R and Graphpad Prism (version 7); protein annotations were extracted from Gene Ontology (GO) and kinase-substrate relations were based on Phosphosite Plus database (October 2019) (phosphosite.org).

Normalized SILAC ratios from the “phospho(STY)sites.txt” file were first loaded in Perseus, log_2_ transformed and the phosphosites were expanded into singly, doubly and multiply phosphorylated sites. Next, the reverse hits and contaminants were filtered out and the dataset was filtered to include at least one valid value. We also removed “normalized H/M” ratios from the dataset which resulted in the identification of 15,898 phosphosites in total. For the hierarchical clustering of all the 15,898 phosphosites, we *Z*-scored the data. The log_2_ values of the replicates per stressor were then median averaged and used for analyses presented. For motif analysis presented in Fig. 3, up- and down-regulated phosphosites were identified by applying a 1.5-fold change cut-off and the numbers are indicated in a table below the figure. For pairwise comparison of the phosphoproteome of the other stressors with that of cisplatin, we compared all six replicates of CDDP to two replicates of each of the other stressors by performing a two-sample Student’s *t*-test with a permutation-based FDR cut-off set at 0.05. Analysis of variance (ANOVA) was performed on the entire dataset consisting of 15,898 phosphosites with a permutation-based FDR cut-off set at 0.05.

### Pathway and motif analyses

For pathway analysis, we used a combination of two criteria to identify stressor-responsive phosphosites being either significantly responding (ANOVA; FDR < 0.05) or passing a fold-change threshold (> 1.5) totaling 7883 phosphosites. These sites were subsequently analyzed using the “Phosphorylation Analysis” module of Ingenuity Pathway Analysis (IPA, Qiagen) software. Significantly responsive pathways were represented using the “Canonical Pathways” module with *p-*value, Benjamini–Hochberg corrected *p-*value and *Z*-score cut-offs set at 0.01, 0.01 and 2, respectively. To identify key kinases and phosphatases upstream of the phosphorylation events, we used the “upstream analysis” module of IPA with *p-*value and *Z*-score cut-offs set at 0.05 and 2, respectively.

Motif analysis was performed using the IceLogo tool (Colaert et al. 2009) using the sequences of the total phosphosites from either individual stressors or the entire phosphoproteome dataset as background depending on the analyses performed. Results were visualized as percentage difference (% difference) and a *p*-value of 0.05 was set as the cut-off.

### Western blot analysis

Western blot analysis of total cell extracts was performed as described previously (Pines et al. 2011) and protein bands were analyzed and visualized with the Odyssey infrared imaging system (LI-COR) using secondary antibodies labeled with visible fluorophores (LI-COR, IRDye^®^ 800CW Goat anti-Mouse IgG (H + L), Catalog No. 926-32210; IRDye^®^ 800CW Goat anti-Rabbit IgG (H + L), Catalog No. 926-32211; IRDye^®^ 680RD Goat anti-Mouse IgG (H + L), Catalog No. 926-68070; and IRDye^®^ 680RD Goat anti-Rabbit IgG (H + L), Catalog No. 926-68071). The primary antibodies employed were mouse anti-phospho-Histone H2AX (Ser139) antibody, clone JBW301 (Millipore, Catalog No. 05-636), rabbit anti-H2B (Millipore, Catalog No. 07-371), mouse anti-pS1981 ATM (10H11.E12) (Cell Signaling Technology, Catalog No. 4526), rabbit anti-ATM (D2E2) (Cell Signaling Technology, Catalog No. 2873), and rabbit anti-pS15 p53 (Cell Signaling Technology, Catalog No. 9284).

### Flow cytometry analysis

For cell cycle analysis, samples were either treated with a stressor for 0.5, 2, 4, or 8 h or mock-treated after which 5-ethynyl-2′-deoxyuridine (EdU, 40 µM in DMSO) was added to the medium. Cells were collected 30 min after the addition of EdU label and stained using the Click-iT^®^ EdU Alexa Fluor^®^ 488 Flow Cytometry Assay Kit (Invitrogen, Catalog No. C-10425) according to the manufacturer's protocol. In brief, 6 × 10^5^ cells were seeded in p60 plates 1 day before the experiment. Cells were treated with stressors and 40 µM EdU was added to the medium 30 min before the end of the exposure. Cells were trypsinized, washed once with 1 ml of 1% BSA in PBS, pelleted by centrifugation. Cell pellets were dislodged using 50 µl of Click-iT^®^ fixative (component D), mixed thoroughly and incubated for 15 min at room temperature, protected from light. Cells were next washed with 1 ml of 1% BSA in PBS and pelleted. Cell pellets were dislodged and resuspended in 50 μl of 1x Click-iT^®^ saponin-based permeabilization and wash reagent (prepared by adding 1 volume of component E to 9 volumes of 1%BSA in PBS, pH 7.1–7.4), mixed well and incubated for 15 min. Click-iT^®^ reaction cocktail was prepared as follows: For each sample, 219 µl PBS, 5 µl CuSO_4_ (component F), 1.25 µl Fluorescent dye azide (prepared by adding 130 µl of DMSO to component B) and 25 µl reaction buffer additive (10 × stock was prepared by adding 2 ml deionized water to component G: Click-iT^®^ EdU buffer additive) were mixed. The Click-iT^®^ reaction cocktail was then added to the cell suspension.

### Cell viability and apoptosis/necrosis assays

Mouse ES cells were treated with either vehicle or stressor for 24 h; ATP Lite (Perkin Elmer, Catalog No. 6016943) was subsequently used for the assessment of cell viability according to the manufacturer's instructions. In brief, cells were lysed by adding 50 µl mammalian cell lysis solution per 100 µl cell suspension and shaken in an orbital shaker for 5 min at 700 rpm. Next, 50 µl substrate solution was added to each well and the microplate was put for 5 min in an orbital shaker at 700 rpm. After the adaptation of the plate for 10 min to the dark, luminescence was measured.

Apoptotic cells were identified by Annexin V staining using the Alexa Fluor^®^ 488 annexin V Dead Cell Apoptosis Kit (Invitrogen, Catalog No. V13241) with RNAse treatment as described previously (Pines et al. 2011). In brief, 6 × 10^5^ cells were seeded in p60 plates 1 day before treatment with stressors. After desired incubation times, the medium was collected, cells were washed with PBS and trypsinized. The reaction was stopped by adding the collected medium to the trypsinized cells. Cells were then centrifuged and washed with PBS. Cells were pelleted, PBS discarded and resuspended in 250 µl 1x annexin-binding buffer (diluted from 5x annexin-binding buffer (component C): 50 mM HEPES, 700 mM NaCl, 12.5 mM CaCl_2_, pH 7.4, with deionized water). Next, 60 µl staining solution (5 µl Alexa Fluor^®^ 488 annexin V (component A: solution in 25 mM HEPES, 140 mM NaCl, 1 mM EDTA, pH 7.4, 0.1% bovine serum albumin (BSA)) and 5 µl 20 µg/ml propidium iodide in 50 µl 1x annexin-binding buffer (component B*:* 1 mg/ml (1.5 mM) solution in deionized water) was added to 50 µl cell suspension of each sample and incubated at room temperature for 15 min. Cells were centrifuged for 5 min at 500*g* and resuspended in cold 100 µl annexin-binding buffer. Next, 100 µl 2% formaldehyde in annexin-binding buffer was added and incubated for 10 min on ice. Cells were centrifuged for 5 min at 500*g* and washed with 200 µl 1% BSA in PBS. Cells were resuspended in 100 µl 1% BSA in PBS and 4 µl of RNAse 20 mg/ml and incubated for 15 min at 37 °C. Subsequently, 10 µl of the cell suspension was transferred to well in 96-well plate with 120 µl 1% BSA in PBS in each well and analyzed using a Guava FACS system. The stained cells were analyzed by flow cytometry, measuring the fluorescence emission at 530 nm and 575 nm (or equivalent) using 488 nm excitation. Flow cytometry results were confirmed by visual inspection with a fluorescence microscope using filters appropriate for fluorescein (FITC) and tetramethylrhodamine (TRITC) or Texas Red^®^ dye.

## Results

### Stressor-induced cellular responses

**Fig. 1:**
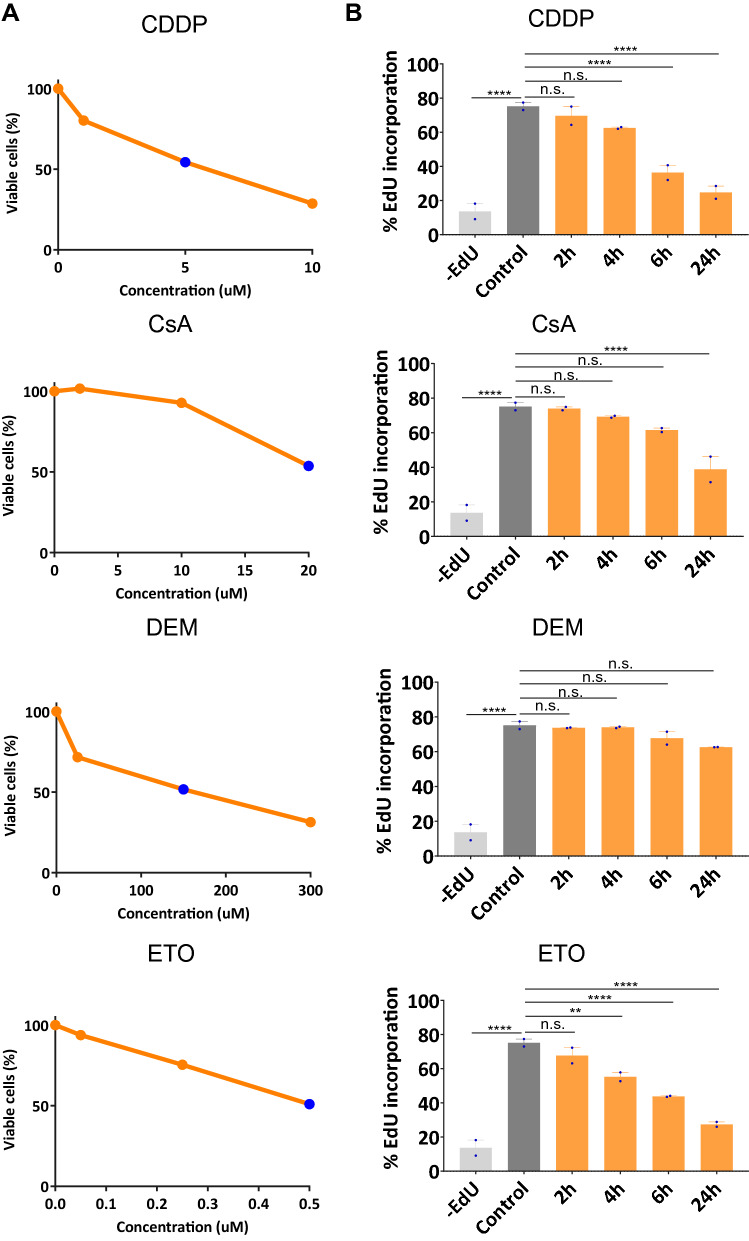
Cellular responses induced by the chemical stressors. **a** mES cells were treated with CDDP, CsA, DEM and ETO and viability was assayed at 24 h after initiation of the treatment by ATP lite at indicated doses. IC50 values are indicated as dark blue dots. **b** Effect of stressors on DNA replication was determined by time course flow cytometric analysis of EdU incorporation. Statistics: one-way ANOVA test between control cells and other indicated treatments (****p-value < 0.001; **p-value = 0.0045; *n.s.* not significant) (colour figure online)

Mouse ES cells are untransformed, can divide indefinitely and have intact DNA damage response pathways and represent an excellent cell system to compare stress responses by chemical agents with different modes of action. We determined the viability of mES cells after 24 h of exposure to the DNA damaging agent CDDP, the topoisomerase inhibitor ETO, the pro-oxidant DEM and the immunosuppressant CsA (Fig. 1a). Inter-stressor comparisons were subsequently made at the dose for each stressor that resulted in approximately 50% reduction of viability (IC50) upon 24 h of treatment as determined by the ATPlite assay. Treatment of mES cells with CDDP and ETO at this equitoxic dose led to an inhibition of DNA synthesis as indicated by a gradual reduction of the EdU incorporation over time (Fig. 1b). Also following CsA exposure, a reduction in EdU incorporation was observed, although less pronounced and delayed when compared to CDDP and ETO treatments. In contrast, EdU incorporation was virtually unaffected by DEM treatment.

### Phosphoproteomics

To identify activation of common and stressor-specific signaling pathways, we performed SILAC-based global phosphoproteomics following exposure to each of the four stressors at the respective IC50 concentration. Cells were grown in SILAC media containing ‘light’, ‘medium’ or ‘heavy’ isotopologues of the amino acids arginine (Arg^0^, Arg^6^, Arg^10^) and lysine (Lys^0^, Lys^4^, Lys^8^) (Supplementary Fig. S1A). In contrast to ion channel and receptor activation-mediated phosphorylation signaling that occurs very early within seconds to minutes time scale (Francavilla et al. 2013; Batth et al. 2018), stress-related phosphorylation signaling cascades are early and intermediate events (Purvis and Lahav 2013). Therefore, we chose 4 h as the time point to profile the phosphoproteome after exposure to each of the four stressors since we aimed to identify common and shared biological pathways. Following trypsinization and cell lysis, SILAC-labeled proteins were mixed in equal ratios and proteolyzed using trypsin. Peptides were first separated into 14 fractions based on their charge using strong cation exchange (SCX) chromatography. Each of the 14 fractions was next enriched for phosphopeptides using TiO_2_ chromatography followed by nano-flow liquid chromatography–tandem mass spectrometry (LC–MS/MS) analysis, quantified using MaxQuant (Tyanova et al. 2016a) and subjected to downstream analysis using Perseus (Supplementary Fig. S1B) (Tyanova et al. 2016b).

We performed, in biological duplicates, three separate experiments in each of which Light- and Heavy-SILAC cells were mock treated and CDDP treated, respectively. The Medium-SILAC cells in each of these experiments were treated with either ETO, CsA, or DEM (Supplementary Fig. 1A). As a result, we obtained six phosphoproteomes each for control and CDDP-treated cells, and two phosphoproteomes each for ETO-, DEM- and CsA-treated cells. This experimental design had three major advantages. First, the inclusion of mock-treated controls in all experiments (6 × L channels) allowed robust SILAC ratio estimations for all stressors over untreated controls. Next, the inclusion of CDDP-treated cells in all experiments (6 × H channels) served as an internal control and increased the statistical power of the dataset enabling the application of false discovery rate (FDR) correction of 0.05 for all the statistical tests. Finally, the inclusion of CDDP-treated cells in all experiments enabled us to perform a comparison of the relative phosphoproteome changes observed after CDDP treatment versus those induced by either ETO, CsA or DEM. We quantified in total 15,898 phosphosites (on 4086 proteins) of which 6667 phosphosites were quantified in all 4 stressors. The phosphoproteome coverage distribution plot (Supplementary Fig. S1C) showed, except for DEM, similar quantification depths for all stressors quantifying 15,884 sites in CDDP-treated, 12,684 sites in CsA-treated, 12,554 sites in ETO-treated and 8324 sites in DEM-treated cells. The experiments showed good reproducibility between biological replicates (Supplementary Fig. S2). The overlap of the global SILAC phosphosite ratio quantifications between two biological replicates was around 65% and is on-par with previously published studies (Hogrebe et al. 2018; Piersma et al. 2015). The distribution of phosphorylated amino acids revealed that the phosphoproteome contained 13,366 (84%) pS, 2425 (15.3%) pT and 107 (0.7%) pY, which is similar to other studies (Olsen et al. 2010; Sharma et al. 2014; Mazouzi et al. 2016). The majority of the phosphosites were localized to a single amino acid [12,746 (80.2%)] with high confidence, Class I sites (phosphopeptide localization probability ≥ 0.75). Of the remainder, 2986 (18.8%) and 166 (1%) phosphosites represented Class II (0.75 > *x* ≥ 0.5) and Class III (0.5 > *x* ≥ 0.25) sites, respectively. Similarly, the distribution of phosphorylation multiplicity revealed that 11,794 (74.2%), 3718 (23.4%) and 386 (2.4%) phosphosites were phosphorylated at single, double or multiple sites, respectively.

**Fig. 2:**
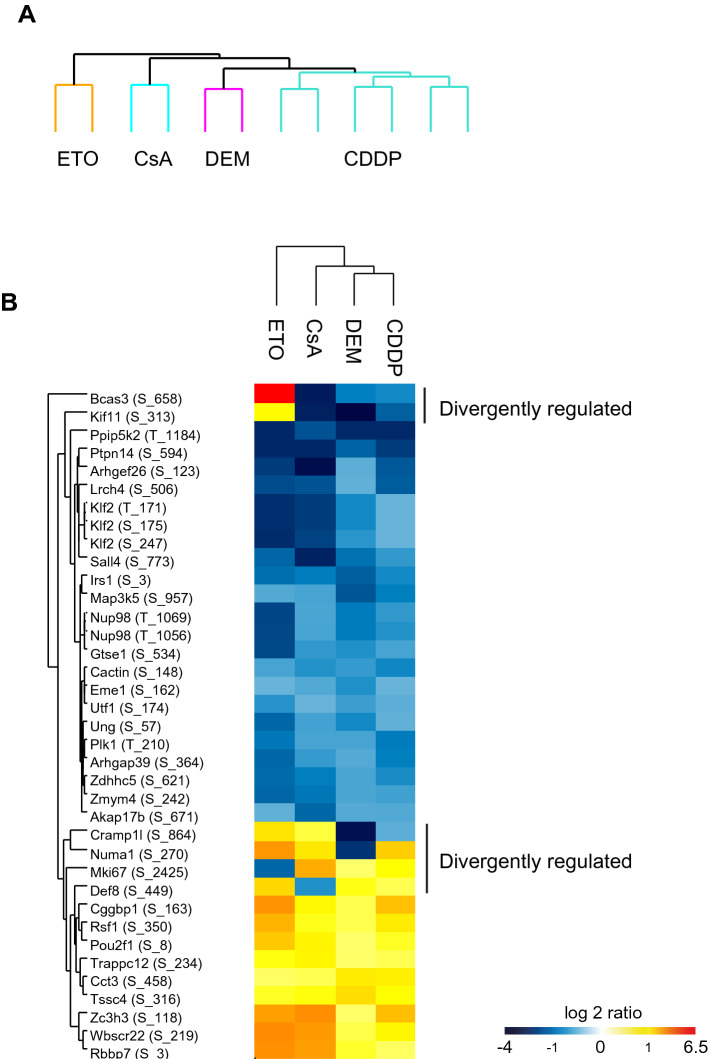
Identification of stressor-specific and stressor-shared differential phosphorylation events. **a** Unsupervised clustering of quantified phosphosites after *z* scoring. **b** Heatmap of 37 responsive phosphosites (> 1.5-fold change) shared by all 4 stressors of which 31 phosphosites responded in the same direction for all 4 stressors

Unsupervised clustering of all 15,898 phosphosites resulted in clear segregation of the stressors (Fig. 2a) with the phosphorylation response after ETO being the most divergent. To compare the phosphorylation responses of the various stressors, we applied a threshold filter of 1.5FC to the averaged data, resulting in 6386 phosphosites (40% of the total sites) that responded to at least 1 of the stressors. We separated these phosphosites based on their direction of response, which resulted in 2677 up-regulated and 3944 down-regulated phosphosites. We found only 37 phosphosites to be commonly responding to all four stressors (Fig. 2b). While 25 of these 37 phosphosites have been reported previously, only one, i.e. pT210 of polo-like kinase1 (*Plk1*) has been described as a regulatory phosphosite. T210 residue is an activation site for *Plk1* and its dephosphorylation is associated with the installment of a cell cycle arrest (Bruinsma et al. 2017). Additionally, we filtered for phosphosites that were quantified in all 12 experiments (6 × reps of CDDP and 2 × reps for each CsA, ETO and DEM). Heatmap visualizations of the global response (Supplementary Fig. S3) and of the top50 upregulated phosphosites (Supplementary Fig. S4) display distinct phosphorylation responses for the different stressors.

### Protein kinase motif analyses

**Fig. 3:**
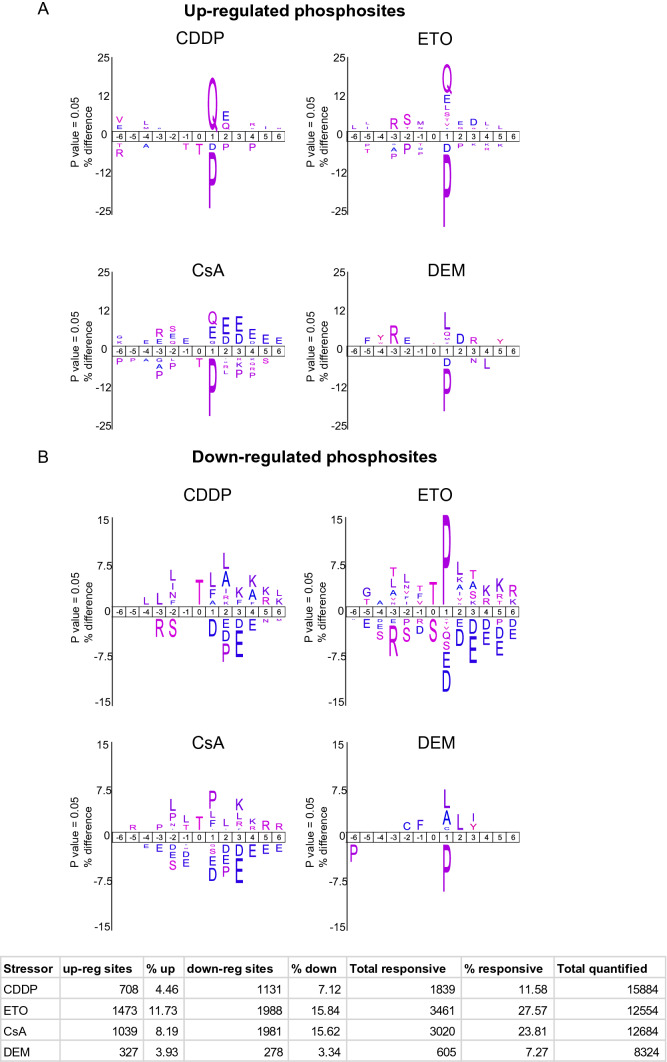
Motif analyses of differentially regulated phosphosites. Sequence motifs analyses and visualization were performed using the IceLogo tool. The total number of phosphosites used to enrich for motifs are indicated per stressor. For each stressor, the sequences of all quantified phosphosites were used as a statistical background. A *p*-value cut-off of 0.05 was applied. The phosphorylated amino acid is located at position 0. **a** Motifs enriched for the indicated stressors among the up-regulated phosphosites. **b** Motifs enriched for the indicated stressors among the down-regulated phosphosites. Table inlet displays an overview of the responsive phosphosites

**Fig. 4:**
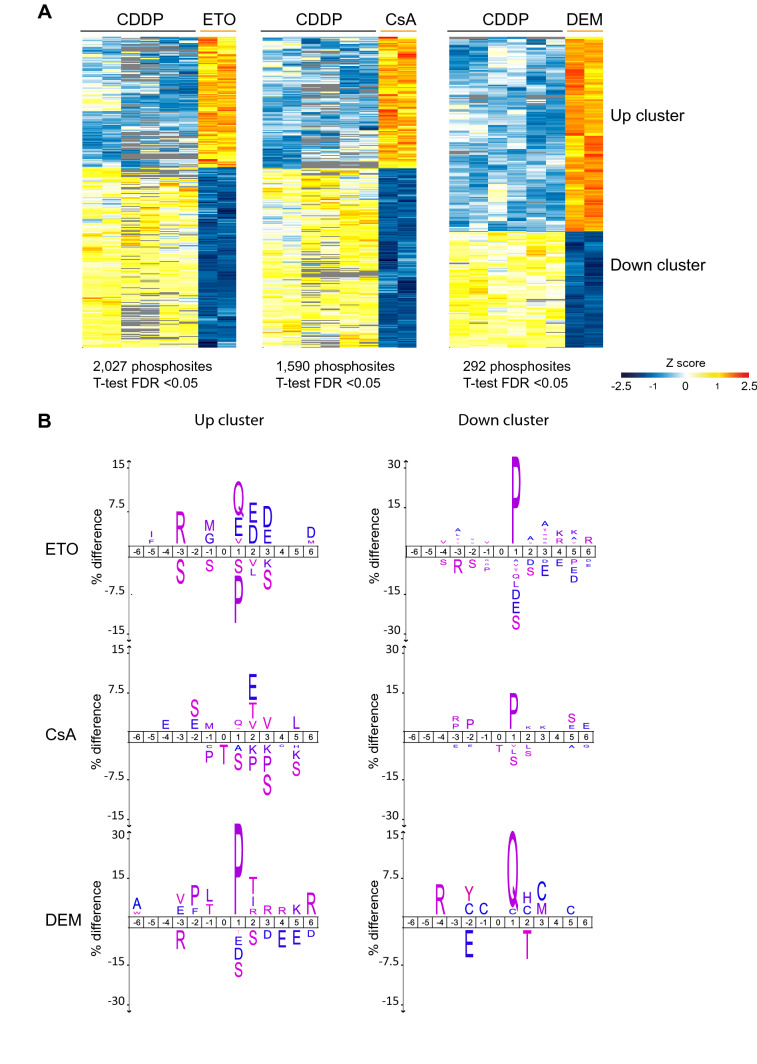
Comparative analysis of the phosphorylation response after ETO, CsA and DEM exposure to the response observed after CDDP treatment. **a** Heatmap of phosphosites that—for the indicated stressors—significantly differed in their regulation compared to CDDP. Clusters with phosphosites that responded more strongly or more weakly compared to CDDP are marked as “up cluster” and “down cluster”, respectively. **b** Motifs enriched for the indicated stressors in “up cluster” (left panel) and “down cluster” (right panel). The sequences of total quantified phosphosites for ETO, CsA or DEM were used as background for the respective cluster. A *p-*value cut-off of 0.05 was applied

To identify upstream protein kinases and phosphatases that are responsible for the global (de)phosphorylation events, we performed motif analysis on the identified stressor-induced phosphosites by applying a threshold filter (1.5-fold change) (Fig. 3). Motif analysis on three biological replicates of CDDP revealed reproducible enrichment of a highly similar kinase motif that was distinctly different from the motifs observed after ETO, CsA, and DEM (Supplementary Fig. S5). CDDP enriched only for the (S/T)Q motif, a consensus motif for the three DDR kinases—*Atm, Atr, and Prkdc,* whereas ETO and CsA enriched apart from the (S/T)Q motif for an acidic residue at + 3 position representing a specificity determinant for *Csnk2a1* (casein kinase II or CK2) kinase (Bian et al. 2013). After CDDP, 19% (136 out of 708) of the upregulated phosphosites were at (S/T)Q sites compared to 12% (180 out of 1473 sites) after ETO, 7% (76 out of 1039 sites) after CsA and 4.5% (15 out of 327 sites) after DEM with the highest phosphorylation amplitudes observed after ETO (Supplementary Fig. S6). Phosphorylated (S/T)Q sites after CsA largely overlapped with those after CDDP and ETO with only three sites being exclusive for CsA. Remarkably, only a few of the identified (S/T)Q sites after CsA (6 out of 76 sites; 7.9%) were on proteins that have been implicated in the DDR in contrast to many more phosphosites following CDDP (40 out of 136 sites, 29.4%) and ETO (39 out of 180 sites, 21.6%) treatments. Analyses of the relative phosphoproteomes of ETO after comparison with CDDP still revealed the (S/T)Q motif for ETO indicating more elevated phosphorylation of substrates of *Atm/Atr/Prkdc* after ETO than CDDP (Fig. 4).

**Fig. 5:**
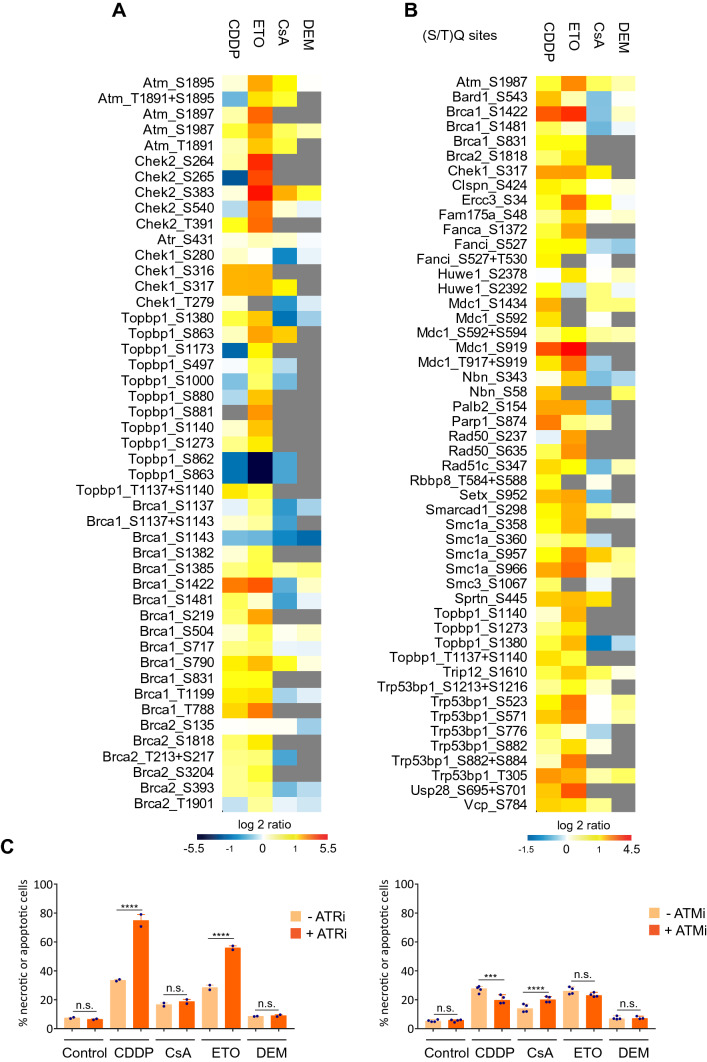
Differential phosphorylation responses of DNA damage response-related proteins. **a** Heatmap of the level of differential phosphorylation at various sites within the DDR-related proteins: Atm, Chek2, Atr, Chek1, Topbp1, Brca1, and Brca2. **b** Heatmap of the level of phosphorylation for 50 phosphosites with an (S/T)Q motif in proteins selected using “DDR” in the “Keywords” annotation from UniProt. **c** Fraction of apoptotic/necrotic cells as determined by Annexin V staining at 24 h after initiation of stressor exposure in the presence or absence of indicated kinase inhibitors. Statistics: one-way ANOVA test between cells treated with indicated stressors with or without the indicated inhibitors (****p-value < 0.001; ***p-value = 0.0006; *n.s.* not significant)

Activation of *Atm* kinase, as indicated by autophosphorylation of S1987 (Fig. 5a), was induced by ETO, to a lesser extent by CDDP and CsA and virtually not by DEM. Interestingly, phosphorylation of *Chek1* at S317, a unique activation marker for the *Atr* kinase, occurred at similar levels after CDDP and ETO treatment, but hardly after exposure to CsA and not after DEM. Unfortunately, the autophosphorylation site of the third DDR-associated kinase *Prkdc*, pS2053, was not detected in our MS experiment and remains undetected by any global MS-based study as evident from the PhosphositePlus database (Hornbeck et al. 2012). Moreover, no unique substrate for the activated *Prkdc* has been identified posing limitations to the interpretation of its involvement in the DDR. Consistent with the relative levels of *Atm* and *Atr* activation, (S/T)Q site phosphorylation of *Rad50* (S237, S635) and *Nbn* (S58, S343) components of the DSB-recognition complex MRN was most pronounced after ETO exposure (Fig. 5b), while *Brca1* and *Brca2*, required for replication fork protection (Schlacher et al. 2012), were equally phosphorylated upon CDDP and ETO exposure but hardly after CsA and not after DEM. The activation of *Atr* requires its interaction with the *Atr*-activator *Topbp1*, which contains an ATR-activation domain that stimulates ATR kinase activity (Duursma et al. 2013). Indeed, we observed distinct phosphorylation of *Topbp1* (S/T)Q sites (pS1140, pS1273 and pS1380) after ETO and CDDP with higher amplitudes after ETO compared to CDDP (Fig. 5b). The abovementioned phosphorylation of components of the DSB-recognition complex MRN by *Atm* activates *Atr* via *Topbp1* recruitment (Duursma et al. 2013; Yoo et al. 2009; Kumagai et al. 2006) leading to *Chek1* activation. In contrast, phosphorylation of *Topbp1* is hardly manifest after CsA and absent after DEM. (Fig. 5b). Western blot analysis confirmed divergent activation of the DDR by the four stressors. Equitoxic doses of CDDP and ETO resulted in a time-dependent increase of the DSB markers γ-H2AX and pS1987 *Atm,* as well as pS15 *Tp53* (indicative of cell cycle arrest and apoptosis) (Supplementary Fig. S7).

In contrast, mES cells exposed to CsA displayed activation of these key nodes of the DDR only after extended exposure time (25 h), while no evidence for the induction of the DDR was observed for DEM. Both ETO and CsA showed, besides enrichment of the (S/T)Q motif, enrichment for an acidic residue at + 3 position suggesting activation of the CK2 kinase by ETO and CsA (Son et al. 2013). Consistently, phosphorylation of several putative substrates of CK2 kinase (Rusin et al. 2017) including *Dkc1* pS453 and *Hdac1* pS423 was enhanced after CsA and ETO (Supplementary Fig. S8). Motif enrichment analysis on the down-regulated phosphosites (Fig. 3b) disclosed a clear enrichment for threonine following treatment with CDDP, ETO or CsA. Threonine-based dephosphorylation is an important mechanism for cell cycle checkpoint activation (Godfrey et al. 2017). Furthermore, enrichment of proline (P) flanking the phosphorylated residue in response to ETO and a lesser extent in response to CsA suggests the inhibition of proline-directed kinases, whereas the enrichment of hydrophobic amino acids leucine (L) and phenylalanine (F) in response to CDDP and a lesser extent after CsA is congruent with diminished Polo-like kinase-mediated phosphorylation (Kettenbach et al. 2012; Nakajima et al. 2003; Cundell et al. 2016; McCloy et al. 2015; Hein et al. 2017; Malik et al. 2009).

Since three of the four stressors prominently induced the activation of *Atm* and/or *Atr* kinases, we wondered whether and to what extent these kinases were involved in the prevention of stressor-induced cell death. To this end, we treated mES cells with the four stressors in the presence and absence of either an *Atm*- or an *Atr*-inhibitor and determined the fraction of apoptotic/necrotic cells. In the absence of kinase inhibitors, apoptosis was induced by CDDP and ETO and to a lesser extent by CsA but not by DEM. While *Atm* inhibition slightly affected apoptosis after CDDP and CsA (Fig. 5c), inhibition *of Atr* lead to an enhanced apoptotic response upon exposure to CDDP or ETO, but not after CsA or DEM, correlating well with the observed extent of *Atr*-mediated phosphorylation of the S317 residue in *Chek1* (Walker et al. 2009). Congruent with the observed extent of replication inhibition (Fig. 1b), these results indicate that the induction of programmed cell death in rapidly proliferating mES cells is predominantly driven by the interference of these stressors with DNA replication.

### Pathway analysis

**Fig. 6:**
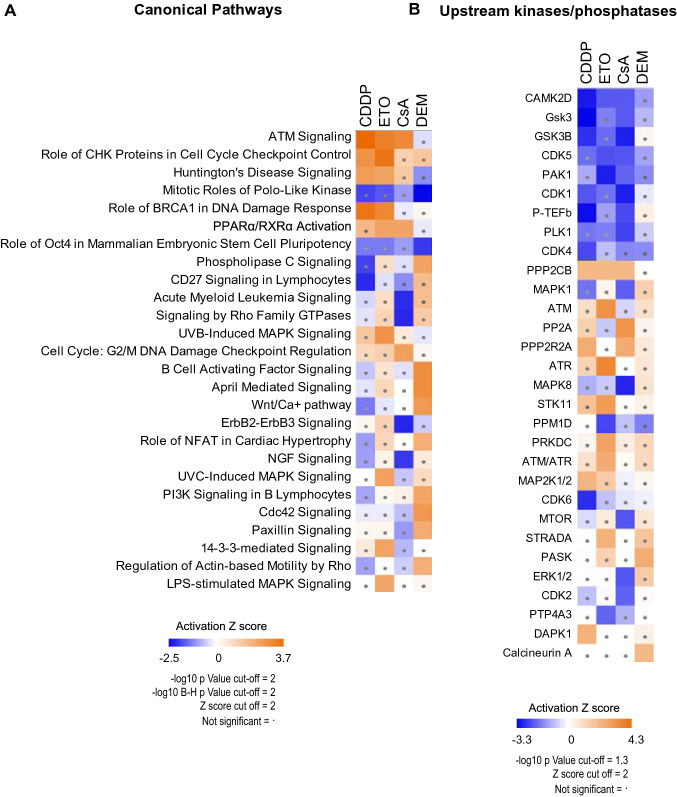
Pathway analysis. **a** Heatmap of the activation *Z* score (IPA) of statistically significantly enriched pathways for at least one of the stressors. Hierarchical clustering was used to group pathways as well as the stressors. Dots in the blocks indicate insignificant values (*z* score < 2). **b** Heatmap of the activation *Z* score (IPA) of statistically significantly enriched upstream kinases and phosphatases for at least one of the stressors. Hierarchical clustering was used to group the kinases/phosphatases as well as the stressors. Dots in the blocks indicate insignificant values (*z* score < 2)

To identify stressor-activated pathways, we extracted the phosphosites that responded to at least one of the four stressors utilizing a combination of threshold filtering and statistical testing. As specified above, the application of a threshold filter of 1.5FC provided us with 6386 responsive phosphosites, while statistical testing (ANOVA test, FDR < 0.05) revealed 3220 phosphosites that responded differentially to all four stressors. Taken together, a total of 7883 stressor-responsive phosphosites was identified out of which 2177 phosphosites are novel sites that have not yet been reported in the PhosphoSitePlus database (October 2019) (Hornbeck et al. 2012). The 7883 stressor-responsive phosphosites were subjected to ingenuity pathway analysis (IPA) to dissect pathways regulated by the four stressors (Fig. 6a). ETO and CDDP share a strong activation of DDR manifested by the significant enrichment of the *Atm*, *Chek1/2*, *Brca1*-related DNA damage signaling, cell cycle checkpoints, and repair pathways. More specifically, ETO-activated pathways included IL-8, 14-3-3 and MAPK-mediated signaling. Only in case of ETO enrichment of upstream kinases, i.e. *Atm*, *Atr*, *Prkdc* (DNA-PKcs), the DDR-related *Stk11* (also known as LKB1) and its activator *Strada* (Fig. 6b) as well as phosphatase *Ppp2cb* (a beta isoform of the catalytic subunit of the PP2A) was statistically significant. CDDP treatment significantly upregulated phosphatase *Ppp2r2a* (also known as B55A), death-associated protein kinase *Dapk1* as well as *Mapk1/2* kinases that are stimulated upon extra- and intracellular signals. Exclusive to CsA is the down-regulation of various signaling pathways (Granzyme B, ErbB2, NGF, Rho GTPase, AML) that are among others involved in the regulation of cell cycle, apoptosis and extracellular matrix degradation (Mishra et al. 2018). Moreover, these pathways are known to be linked to kinases (i.e. MAPK, ERK, CDK) specifically down-regulated after treatment with CsA. Interestingly, activation of the *Atm* signaling pathway was also manifest in CsA-exposed mES cells. DEM exposure significantly activated a wide array of signaling pathways (e.g. Phospholipase C, B-cell activating factor) with upregulation of the nutrient and stress-responsive PAS domain-containing serine/threonine-protein kinase (*Pask*) and the redox-sensitive serine/threonine-dependent phosphatase calcineurin. DEM hardly affected DDR pathways and upstream kinases/phosphatases. On the contrary, DEM was the only stressor that significantly down-regulated pathways controlling mitotic exit (Polo-like kinase-related networks) as well as self-renewal and differentiation (Oct4 related networks), yet no significant changes in upstream kinases *Plk1* or *Cdk1* (regulator of Oct4) (Kim et al. 2018) were manifest. Despite this lack of significance from the upstream analysis of IPA, the down-regulated phosphosites that responded to all stressors (Fig. 2b), included pT210 *Plk1* as the only known regulatory phosphosite. Dephosphorylation of this site leads to the inactivation of *Plk1* and consequently cell cycle arrest (Bruinsma et al. 2017). ETO, CDDP, and CsA shared significant down-regulation of *Gsk3* and *Camk2d* kinases, whereas the DEM-mediated change appeared to be not significant. *Gsk3* and *Camk2d* kinases act as negative regulators of the Wnt pathway (Patel and Woodgett 2017; Carreras Puigvert et al. 2013; Cong et al. 2014). Consistently, only DEM significantly upregulated the Wnt pathway.

## Discussion

Here, we present a global phosphoproteome analysis of mES cells exposed to equitoxic doses (50% cell viability) of four stressors with distinct modes of action that do not require bioactivation. Over 7883 stressor-responsive phosphosites were identified. The four stressors elicit cellular responses through distinctive and complex phosphorylation signaling cascades involving different kinases and phosphatases. However, the global activation of the kinases (and phosphatases), as well as functional interpretation of the phosphoproteome dataset is vastly restricted by the small proportion of reported phosphosites (~ 2%) that have been assigned to a regulatory kinase and another small proportion (~ 3%) that have a reported biological function. The fact that many of these phosphosites are substrates of more than one kinase further complicates data interpretation (Needham et al. 2019). While ETO (~ 28%) and CsA (~ 24%) displayed strong global phosphoproteome changes based on threshold filtering, CDDP (~ 12%) and DEM (~ 7%) displayed moderate and weak phosphoproteome changes, respectively. Motif and pathway analyses did not reveal any common motifs and pathways for all four stressors.

Analysis of the up-regulated phosphosites showed that ETO exposure led to activation of *Atm* as well as *Atr* as evident from extensive phosphorylation of their downstream effectors *Chek2* (5 sites) and *Chek1* (2 sites), respectively. In contrast, after CDDP, only *Atm* S1987 was significantly phosphorylated. CDDP-mediated phosphorylation of *Atr*-dependent *Chek1* (2 sites) was qualitatively and quantitatively similar to ETO. The picture that emerges is that both DNA damage-inducing agents generate replication-blocking lesions leading to similar levels of *Atr* activation, whereas the differential activation of *Atm* appears to reflect differences in the rate at which DSBs are being generated by ETO and CDDP or possibly their nature. ETO can induce replication-independent DSB resulting from the collision of the transcription machinery with trapped topoisomerase II, while replication-blocking lesions induced by either ETO or CDDP will predominantly give rise to DSBs during the (next) S-phase and thus appear at later time points than 4 h after initiation of treatment. Indeed, *Atm* auto-phosphorylation at S1987 as determined by western blot analysis displayed more rapid kinetics after ETO than CDDP exposure (Supplementary Fig. S7). In line with the phosphorylation data indicating activation of cell cycle checkpoints related to replication stress, treatment with ETO and CDDP-induced time-dependent inhibition of DNA synthesis as well as apoptosis as reported previously (Lehman et al. 2017; Brozovic et al. 2009; Tammaro et al. 2013; Wu et al. 2011). The apoptotic response in mouse ES cells appears to be predominantly driven by replication stress as inhibition of *Atr* but not *Atm* enhanced the induction of apoptosis. Pathway analysis also indicated a strong regulation of several DDR-related pathways after ETO and/or CDDP including mitogen-activated protein kinase (MAPK), CHEK and BRCA1-mediated pathways.

The CsA phosphoproteome also showed enrichment of the (S/T)Q motif but with a prominent enrichment for an acidic residue at + 3 position indicative of enhanced CK2 activity (Bian et al. 2013). CsA-associated phosphorylation of *Atm* (four sites including S1987 and T1891), *Chek1* (one site) and *Chek2* (one site) coincide with a decrease in DNA synthesis albeit significantly delayed when compared to ETO and CDDP. Different from ETO and CDDP, the apoptotic response after CsA appeared not to be affected by *Atr* inhibition. Whereas western blot analysis could not demonstrate active *Atm* signaling at 4 h after CsA, a moderate increase of y-H2AX and pS1987 *Atm* levels was observed after 24 h exposure suggestive of the induction of DSBs. It is tempting to speculate that the observed inhibition of DNA synthesis and apoptosis induction is related to a delayed generation of DSBs by CsA treatment. Indeed, exposure of primary human skin fibroblasts to a low dose of CsA (5 μM) during 24 h has been shown to induce *Tp53bp1* foci (indicative for DSBs). These breaks have been suggested to arise from SSB during DNA replication and were suggested to result from a general inability to repair DSBs (Zhang et al. 2006). Specific CsA responses at the phosphoproteome level include the down-regulation of pathways that control hematopoietic differentiation and immunity (Granzyme B, ErbB2, NGF, Rho GTPase, AML) being consistent with known effects of CsA as an immunosuppressant (Zhou et al. 2015) and inducer of apoptosis (Koppelstaetter et al. 2018).

We did not observe any indication that DNA damage signaling was activated after DEM, consistent with reports that the induction of the DNA damage response by DEM is only observed after high concentrations (Hiemstra et al. 2017). Rather, DEM-associated stress pathways appear to be linked to processes responding to enhanced levels of oxidative stress in line with its accepted mode of action as pro-oxidant. One of the pathways activated after DEM exposure concerns the induction of *Pask* and the redox-sensitive serine/threonine-dependent phosphatase calcineurin. *Pask* is a regulator of intracellular signaling pathways responding to both extrinsic and intrinsic stimuli and is important for the proper regulation of glucose metabolism and oxidative stress in mammals (Huang et al. 2014).

Among down-regulated phosphosites, a clear enrichment for threonine was observed after exposure to CDDP, ETO, and CsA, but not to DEM. This observation correlates well with the observed levels of DNA replication inhibition as threonine-based phosphorylation signaling is especially associated with cell cycle progression and its dephosphorylation is an important mechanism for cell cycle checkpoint activation (Godfrey et al. 2017). Dephosphorylation of threonine could be mediated by phosphatases targeting sites that are substrates of proline—or hydrophobic residue—directed kinases consistent with the overrepresentation of proline (ETO and CsA) and leucine and phenylalanine (CDDP and CsA) in the downregulated phosphosites. The phosphatase Pp2A-B55 has been shown to inhibit proline-directed phosphorylation specifically against threonine/proline motifs conferring time resolution in cell cycle checkpoint activation and progression (McCloy et al. 2015; Hein et al. 2017). Motif analysis indicates that the checkpoint might be activated by Pp2A-related phosphatase activities notably Pp2A-B55. It has been shown that PP2A can induce apoptosis (Zhou et al. 2017) and counteract *Atr-Chek1* signaling (Leung-Pineda et al. 2006).

In summary, the DNA damaging agent CDDP, the topoisomerase inhibitor ETO, the immunosuppressive agent CsA and the pro-oxidant DEM elicit distinctive and complex phosphorylation signaling cascades at equitoxic doses. Phosphoproteome analyses revealed stressor-specific pathways and kinase motifs related to their cellular actions. This study demonstrates that phosphoproteomics analysis is a powerful tool to characterize and dissect the various signaling responses after exposure to different types of stress. The various types of stressors displayed substantial differences in their signaling responses even in the case of CDDP and ETO that both generate replication stress and induce apoptosis via DNA damage.

## Electronic supplementary material

Below is the link to the electronic supplementary material.

Supplementary file1 (PDF 891 kb)
