# Supplementary material for: Quantitative phosphoproteomics to unravel the cellular response to chemical stressors with different modes of action
Source: Arch Toxicol. 2020 Mar 18;94(5):1655–71. doi: 10.1007/s00204-020-02712-7 (PMC7261734; doi:10.1007/s00204-020-02712-7)
Supplement: Supplementary file 1 — Supplementary file1 (PDF 891 kb) [file 204_2020_2712_MOESM1_ESM.pdf]

Fig. S1

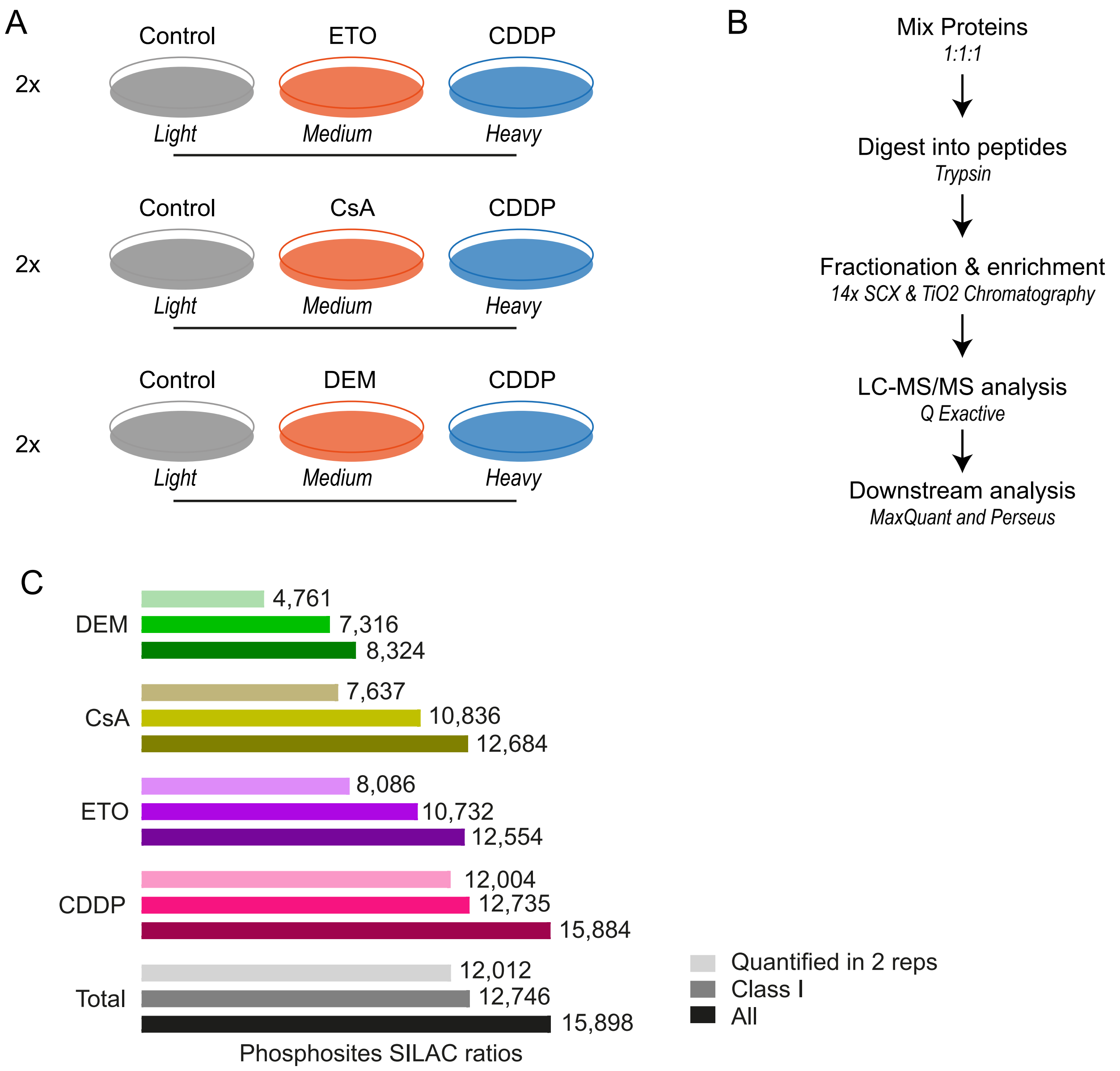
A
Control
ETO
CDDP
2x
Light
Medium
Heavy
Control
CsA
CDDP
2x
Light
Medium
Heavy
Control
DEM
CDDP
2x
Light
Medium
Heavy
B
Mix Proteins
1:1:1
Digest into peptides
Trypsin
Fractionation & enrichment
14x SCX & TiO2 Chromatography
LC-MS/MS analysis
Q Exactive
Downstream analysis
MaxQuant and Perseus
C
DEM
4,761
7,316
8,324
CsA
7,637
10,836
12,684
ETO
8,086
10,732
12,554
CDDP
12,004
12,735
15,884
Total
12,012
12,746
15,898
Phosphosites SILAC ratios
Quantified in 2 reps
Class I
All

Fig. S2

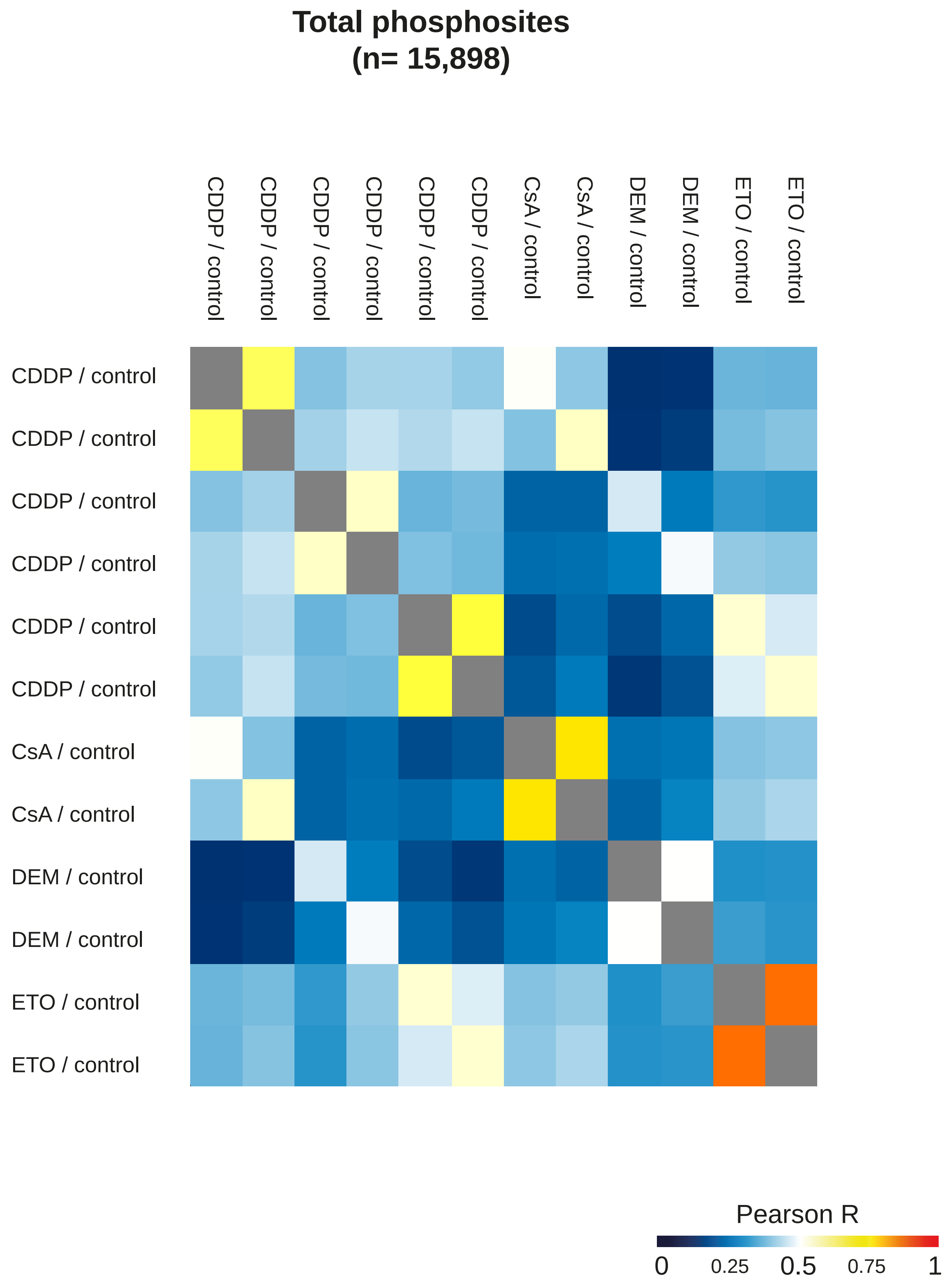
Total phosphosites
(n= 15,898)
CDDP / control
CDDP / control
CDDP / control
CDDP / control
CDDP / control
CDDP / control
CsA / control
CsA / control
DEM / control
DEM / control
ETO / control
ETO / control
Pearson R
0
0.25
0.5
0.75
1

Fig. S3

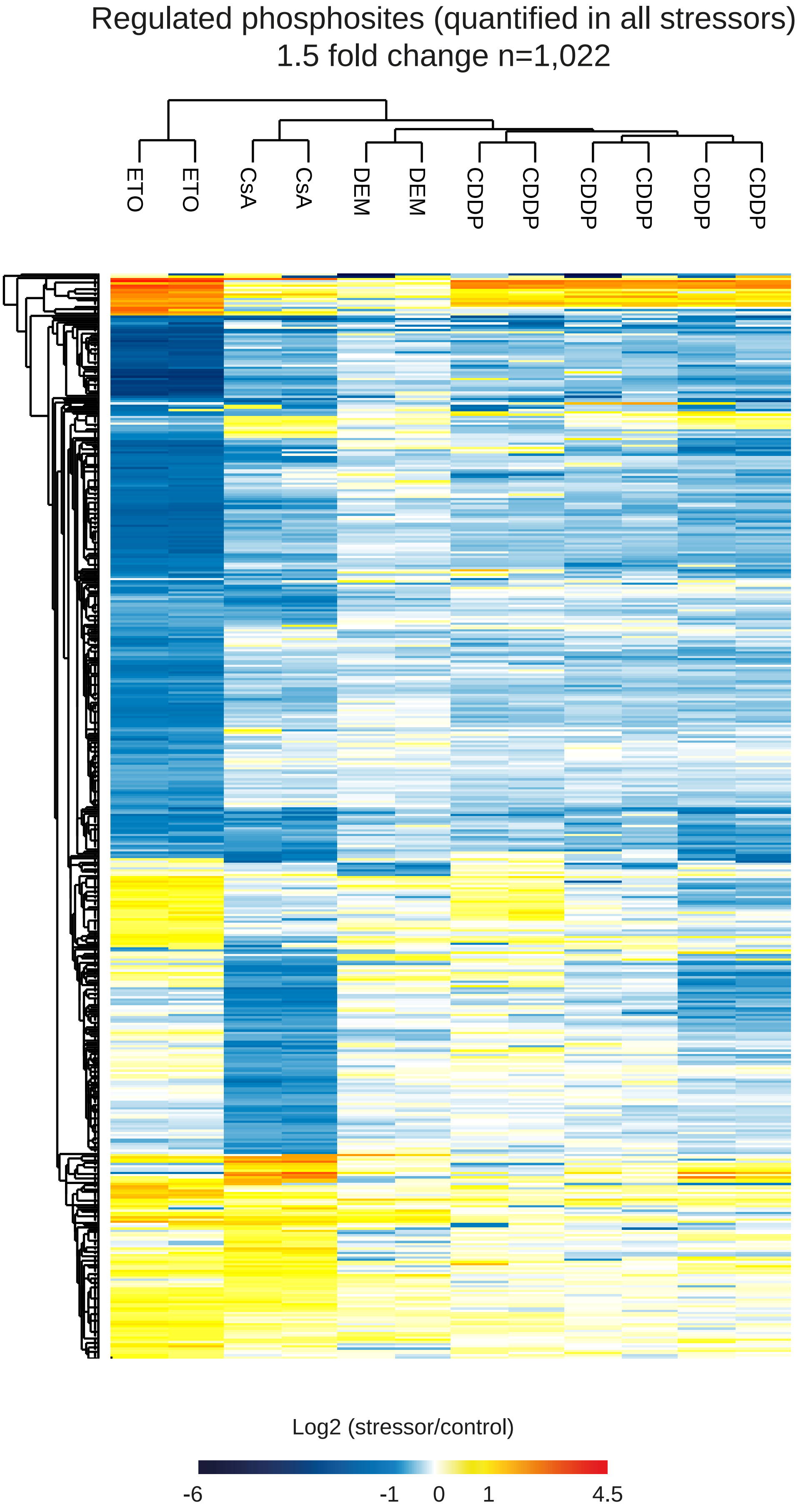

Regulated phosphosites (quantified in all stressors)
1.5 fold change n=1,022
ETO
ETO
CsA
CsA
DEM
DEM
CDDP
CDDP
CDDP
CDDP
CDDP
CDDP
Log2 (stressor/control)
-6
-1
0
1
4.5

Fig. S4

Top 50 up-regulated phosphosites

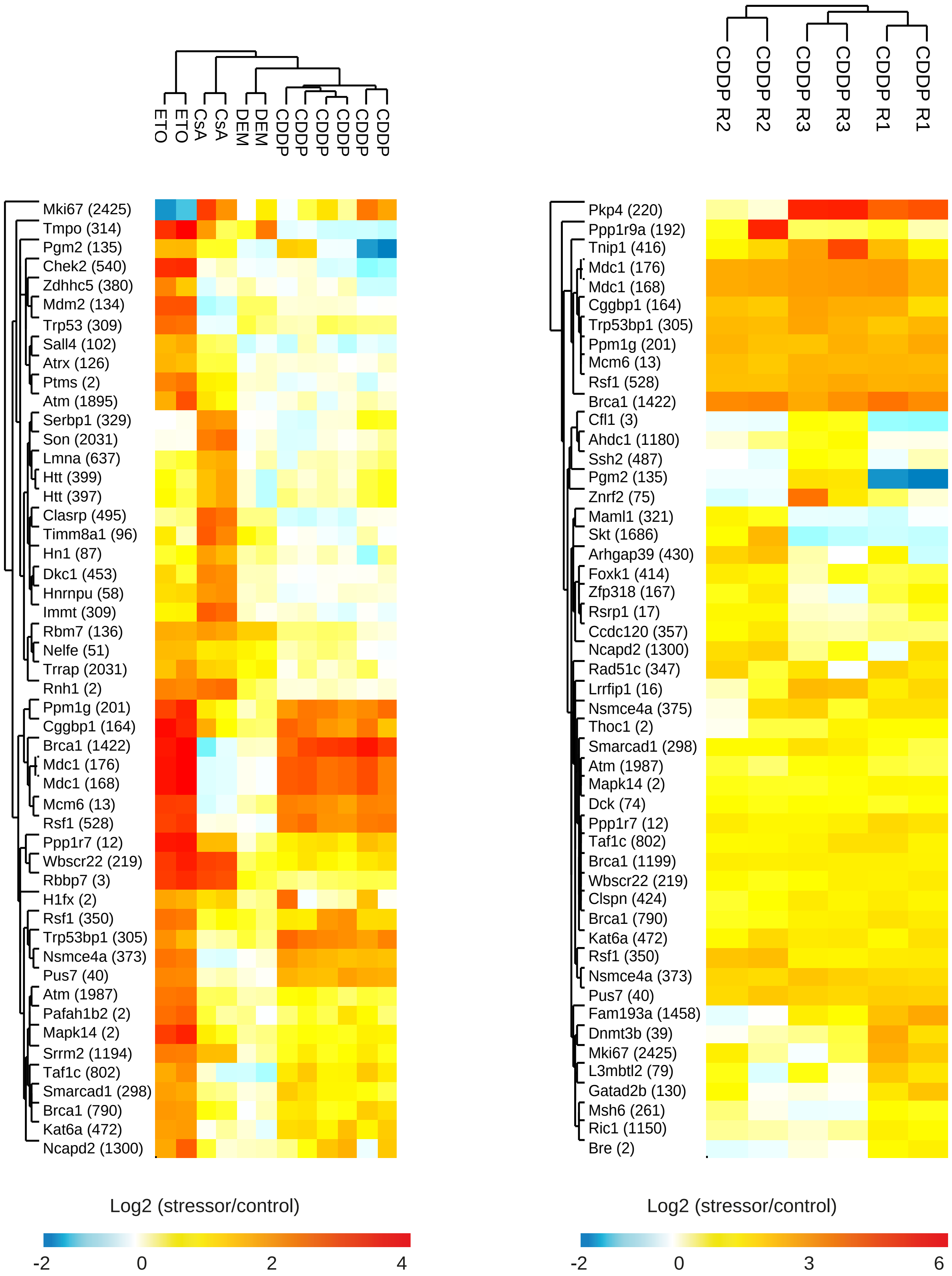

Fig. S5

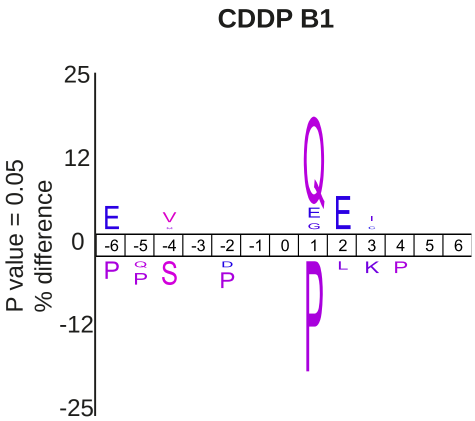
CDDP B1
P value = 0.05
% difference
25
12
0
-12
-25
-6 -5 -4 -3 -2 -1 0 1 2 3 4 5 6

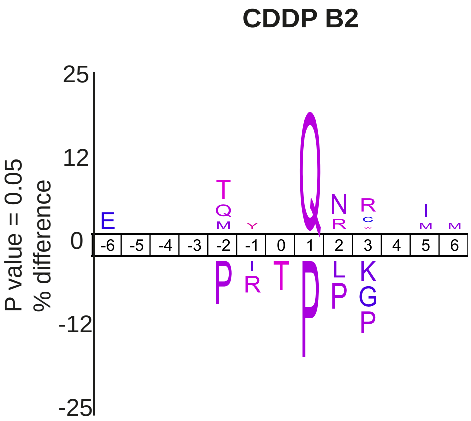
CDDP B2
P value = 0.05
% difference
25
12
0
-12
-25
-6 -5 -4 -3 -2 -1 0 1 2 3 4 5 6

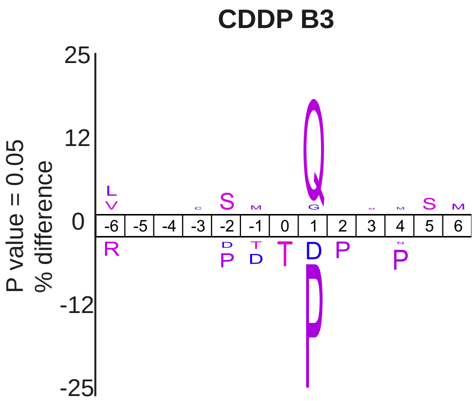
CDDP B3
P value = 0.05
% difference
25
12
0
-12
-25
-6 -5 -4 -3 -2 -1 0 1 2 3 4 5 6

Fig. S6

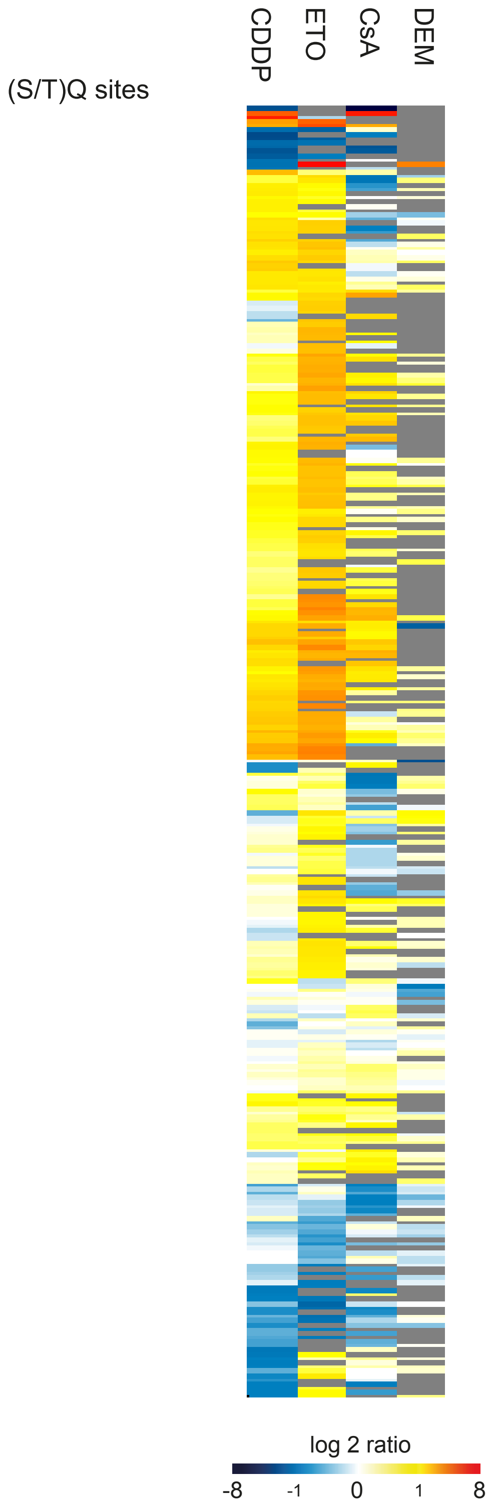

Stressor	up-reg sites	up-reg p(S/T)Q sites	up-reg p(S/T)Q sites (%)
CDDP	708	136	19%
ETO	1473	180	12%
CsA	1039	76	7%
DEM	327	15	4.5%

Fig. S7

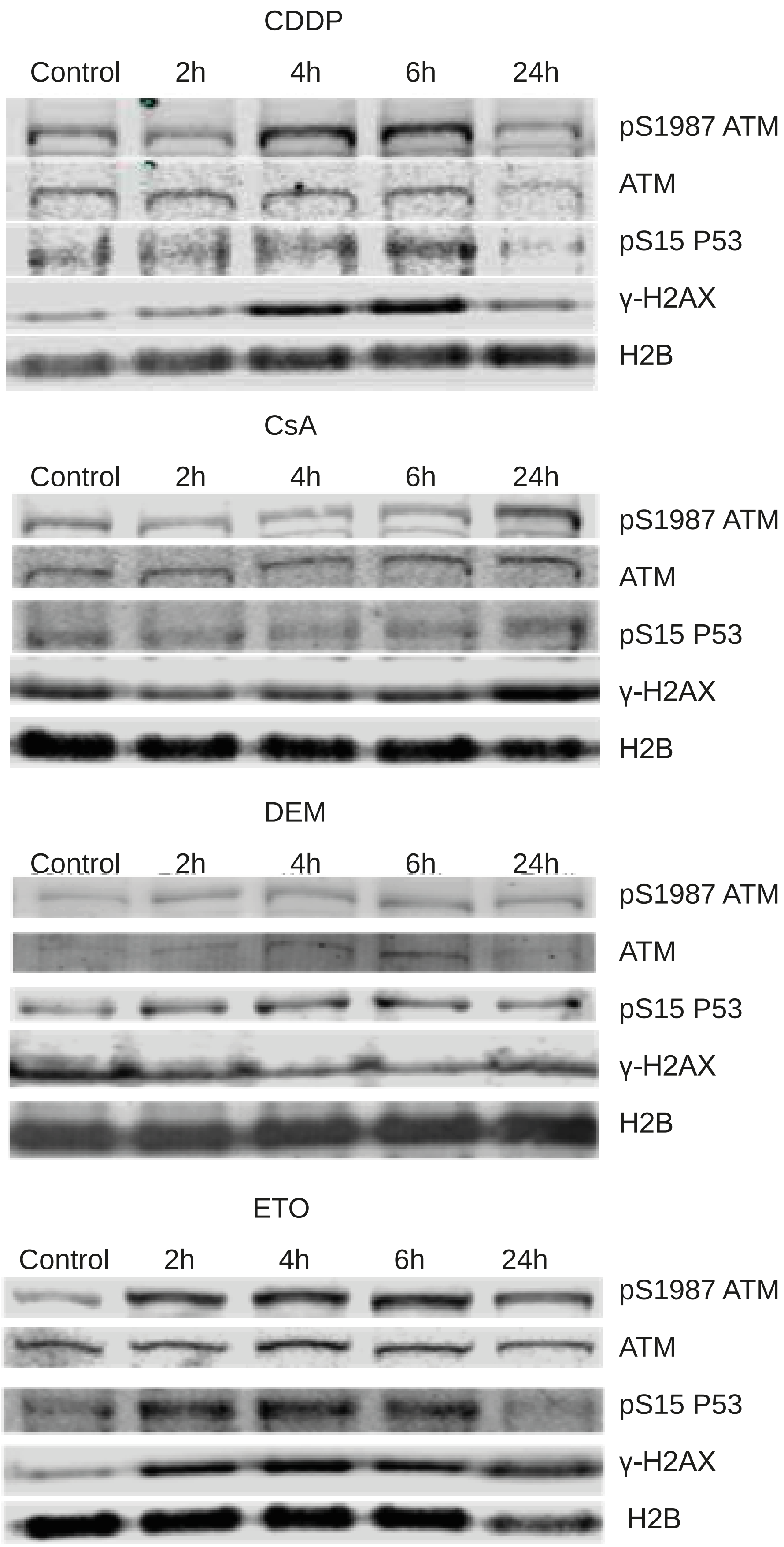
CDDP
Control
2h
4h
6h
24h
pS1987 ATM
ATM
pS15 P53
γ-H2AX
H2B
CsA
Control
2h
4h
6h
24h
pS1987 ATM
ATM
pS15 P53
γ-H2AX
H2B
DEM
Control
2h
4h
6h
24h
pS1987 ATM
ATM
pS15 P53
γ-H2AX
H2B
ETO
Control
2h
4h
6h
24h
pS1987 ATM
ATM
pS15 P53
γ-H2AX
H2B

Fig. S8

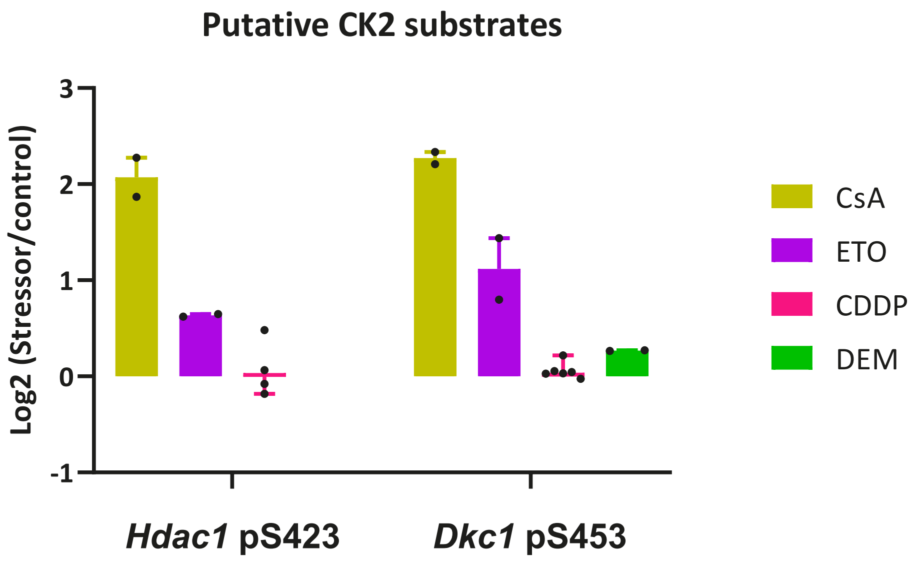

Putative CK2 substrates
Log2 (Stressor/control)
3
2
1
0
-1
Hdac1 pS423
Dkc1 pS453
CsA
ETO
CDDP
DEM

Supplementary figure legends

Figure S1. Stressor-induced phosphoproteomes. (A) Experimental setup. In all three experiments (performed in biological duplicates) Light SILAC and Heavy SILAC cells were mock-treated and CDDP-treated, respectively. Medium SILAC cells were treated with either ETO, CsA or DEM. (B) Step-by-step phosphoproteomics workflow with specific methodology indicated in italics. (C) Coverage of the global phosphoproteome indicating the number of phosphosites quantified for each experiment.

Figure S2. Heatmap shows Pearson R values of log2 phosphosite SILAC ratios (stressors over control) of all quantified 15,858 phosphosites.

Figure S3. Heatmap showing the log2 phosphosite SILAC ratios over untreated controls that are quantified in all four stressors (including all replicates) that passed a 1.5-fold change cut-off.

Figure S4. Heatmap showing log2 phosphosite SILAC ratios of top 50 up-regulated phosphosites that are quantified in all four stressors including all replicates (left panel) and that are quantified in all six CDDP replicates (right panel).

Figure S5. Motif analyses of differentially regulated phosphosites for each biological replicate of CDDP. Sequence motifs analyses and visualization was performed using the IceLogo tool. For each replicate, the sequences of all quantified phosphosites were used as a statistical background. A p-value cut-off of 0.05 was applied. The phosphorylated amino acid is located at position 0. Motifs enriched for the indicated replicate of CDDP among the up-regulated phosphosites.

Figure S6. Heatmap showing the log2 phosphosite SILAC ratios over untreated controls of phosphosites with (S/T)Q motif that responded to any of the stressors. **Table inlet** displays an overview of the fraction of up-regulated (S/T)Q phosphosites.

Figure S7. Western blot probing dynamic changes of pS1987 ATM, ATM, pS15 p53, γ-H2AX and H2B after treatment with CDDP (top panel), CsA (second panel from top), DEM (second panel from bottom) and ETO (bottom panel)

Figure S8. Bar chart of site-specific quantifications of two example putative CK2 substrates.
